# Special Issue: “New Insights of Biomarkers in Neurodegenerative Diseases”

**DOI:** 10.3390/ijms262210869

**Published:** 2025-11-09

**Authors:** Ioannis Liampas

**Affiliations:** Department of Neurology, Faculty of Medicine, School of Medicine, University of Thessaly, Mezourlo Hill, 41100 Larissa, Greece; iliampas@uth.gr

## Editorial for the Special Issue “New Insights of Biomarkers in Neurodegenerative Diseases”

The early and accurate detection and monitoring of neurodegenerative disorders remain among the most pressing challenges in modern neuroscience [1,2]. Biomarkers—objectively measurable indicators of biological processes or pathological changes—play a crucial role in disease identification, patient stratification, prognosis, and monitoring of therapeutic responses [3,4]. This Special Issue aimed to consolidate and expand the current evidence on molecular biomarkers for neurodegenerative diseases.

In neurodegenerative disorders such as Alzheimer’s disease (AD) and frontotemporal dementia (FTD), clinical symptoms typically emerge after substantial neuronal loss has already occurred [5,6]. Even in full-blown clinical syndromes, considerable phenotypic overlap often complicates accurate differential diagnosis [7,8,9]. Thus, early and reliable detection using sensitive biomarkers is essential. Traditional imaging (such as dopamine transporter scans) and cerebrospinal fluid (CSF) biomarkers [such as amyloid-β (Aβ) and phosphorylated tau (p-tau)] have provided valuable diagnostic insights [10,11]. Nonetheless, the field is now moving rapidly toward the development of minimally invasive and cost-effective biomarkers that are suitable for large-scale screening and routine clinical application [12,13].

The advent of ultrasensitive assay technologies—such as single-molecule array (Simoa), immunoprecipitation–mass spectrometry, and electrochemiluminescence platforms—has enabled reliable quantification of low-abundance proteins in plasma, facilitating the emergence of blood-based biomarkers in AD [14,15,16,17,18]. Among these, p-tau species (p-tau181, p-tau217, p-tau231) have demonstrated strong correlations with amyloid and tau PET imaging, as well as cerebrospinal fluid (CSF) biomarkers, accurately discriminating AD from other neurodegenerative dementias [19,20,21]. Neurofilament light chain (NfL), a marker of axonal injury, shows associations with disease progression and cognitive decline across the AD continuum [22,23,24]. Glial fibrillary acidic protein (GFAP) and soluble triggering receptor expressed on myeloid cells 2 (sTREM2) provide insights into astroglial and microglial activation, respectively [25,26,27,28]. Ongoing efforts are directed toward defining assay-specific reference ranges, harmonizing preanalytical protocols, and evaluating biomarker performance across diverse populations and comorbidities [29,30,31]. These initiatives collectively aim to establish blood-based biomarkers as accessible, scalable tools for diagnosis, prognosis, and treatment monitoring in clinical and research settings in AD.

Despite significant progress, current biomarkers for neurodegenerative diseases face several important limitations [32]. Precision and reproducibility remain challenging: for example, plasma and CSF levels of Aβ and p-tau can vary with age and comorbidities [33,34]. Standardization across laboratories is incomplete, complicating interpretation and cross-study comparison. Cost and accessibility are also barriers, especially for sophisticated imaging modalities or advanced multi-omics assays, limiting their widespread application. Finally, most biomarkers capture a single pathological dimension, failing to reflect the multifactorial nature of neurodegeneration and underscoring the need for integrated multi-marker panels. These limitations highlight the necessity for continued research to validate and standardize existing markers, as well as to identify novel targets. This Special Issue features five articles that span human and animal studies and theoretical perspectives on biomarkers in neurodegenerative disorders.

Martínez-Dubarbie and colleagues analyzed cognitively unimpaired participants to explore potential determinants of plasma amyloid and tau levels. The authors demonstrated that renal function significantly affects plasma Aβ40 and Aβ42 levels, but not the Aβ42/Aβ40 ratio. The glomerular filtration rate (GFR) exhibited an inverse association with plasma Aβ40 and Aβ42, but not with the Aβ42/Aβ40 ratio. The amyloid ratio was lower in individuals with diabetes mellitus and hypertension, while p-tau-181 was higher in cognitively healthy adults with hypertension. These findings emphasize the importance of considering renal and cardiovascular comorbidities when interpreting biomarkers and suggest potential differential applications in distinct patient populations [35].

Sampatakakis and colleagues investigated whether physical function is related to CSF biomarkers in individuals along the AD continuum. The authors revealed that measures of physical function—such as gait speed and hand-grip strength—correlate with CSF Aβ42 in individuals along the AD continuum, an association which is stronger among those with MCI than in those without MCI. These findings expand our current knowledge regarding the physical function parameters in the AD biological continuum and bridge molecular pathology with physical performance in AD [36].

Filippenkov and colleagues studied gene expression patterns in animal brains with ischemic insults. Ischemic injury led to overlapping and differential spatial regulation of gene expression in striatum and frontal samples. Upregulated genes were related to glutamatergic, immune-related, and apoptotic pathways, among others, while downregulated genes were linked to pathways associated with neurotransmission. These findings may help identify both harmful and regenerative processes in brain cells following ischemic injury. Given the critical role of vascular insults in neurodegenerative diseases, these transcriptomic insights could also have important implications for understanding and potentially targeting neurodegenerative processes [37].

Volloch and Rits-Volloch present a theoretical framework proposing a pivotal role for intracellular Aβ accumulation as driving Alzheimer’s disease pathogenesis. This perspective extends their earlier theoretical papers, which argued that conventional models centered on extracellular amyloid deposition fail to explain disease progression or therapeutic failures. While their hypothesis is intellectually provocative and mechanistically plausible, it remains largely speculative; supporting data are primarily conceptual or indirect, and independent empirical validation has yet to be demonstrated [38].

Finally, Crescenzo and colleagues present a case of chronic graft-versus-host disease manifesting with neurodegenerative-like changes. A 65-year-old man treated with hematopoietic stem cell transplant presented progressive visual loss and physical and cognitive deterioration. This case highlights that neurodegenerative-like changes may arise in unexpected clinical settings, and biomarkers may help differentiate between neurodegenerative and potentially treatable disorders [39].

Collectively, these contributions advance the field by integrating molecular, physiological, and theoretical perspectives, while stressing the ongoing need for validation, standardization, and clinical translation in biomarker research. Future studies should focus on harmonizing plasma and CSF biomarkers across age, sex, ethnic background, and comorbidity profiles. In addition, investigations of novel biomarker classes and multi-modal panels may identify markers with enhanced diagnostic or prognostic value. Finally, the potential application of current or emerging biomarkers as surrogate endpoints in clinical trials or for guiding precision medicine approaches warrants further exploration [4,40,41].
